# Sarcopenia in Hospitalized Older Adults: A Cross-Sectional Study Comparing Diagnostic Thresholds and Handgrip Strength Measurement Tools

**DOI:** 10.3390/geriatrics11010007

**Published:** 2026-01-04

**Authors:** Eliana Hanna-Deschamps, François R. Herrmann, Diana Chirouzes, Laurence Claudepierre Buratti, Christophe Luthy, Emilia Frangos, Sophie Pautex, Laurence Genton, Dina Zekry, Christophe E. Graf, Aline Mendes

**Affiliations:** 1 Division of Internal Medicine and Rehabilitation Beau-Séjour, Department of Rehabilitation and Geriatrics, University Hospitals of Geneva, 1205 Geneva, Switzerland; eliana.hanna@hug.ch (E.H.-D.); christophe.luthy@hug.ch (C.L.); 2 Division of Geriatrics and Rehabilitation, Department of Rehabilitation and Geriatrics, University Hospitals of Geneva, 1226 Geneva, Switzerland; francois.herrmann@hug.ch (F.R.H.); diana.chirouzes@gmail.com (D.C.); laurence.claudepierre@hug.ch (L.C.B.); christophe.graf@hug.ch (C.E.G.); 3 Division of Rehabilitation Loëx/Joli-Mont, Department of Rehabilitation and Geriatrics, University Hospitals of Geneva, 1233 Geneva, Switzerland; emilia.frangos@hug.ch; 4 Division of Palliative Care, Department of Rehabilitation and Geriatrics, University Hospitals of Geneva, 1205 Geneva, Switzerland; sophie.pautex@hug.ch; 5 Clinical Nutrition, Department of Medicine, University Hospitals of Geneva, 1211 Geneva, Switzerland; laurence.genton@hug.ch; 6 Division of Internal Medicine for the Aged, Department of Rehabilitation and Geriatrics, University Hospitals of Geneva, 1226 Geneva, Switzerland; dina.zekry@hug.ch

**Keywords:** sarcopenia, muscle mass, fat-free mass index, handgrip strength, dynamometer, vigorimeter

## Abstract

Background: Sarcopenia is highly prevalent among hospitalized older adults and is associated with poor clinical outcomes. Multiple diagnostic criteria exist, but the comparative implications of different handgrip strength (HGS) thresholds and measurement tools are less explored. Objectives: This study aimed to assess the prevalence of sarcopenia, comparing the diagnostic yield of different HGS thresholds using two measurement instruments (dynamometer and vigorimeter) in hospitalized older adults. Design: This was a cross-sectional observational study. Setting: A tertiary geriatric hospital with acute, rehabilitation, and long-term care wards was included. Participants: A total of 376 hospitalized older adults with complete HGS and bioelectrical impedance analysis (BIA) data were recruited. Measurements: HGS was measured using both a hydraulic dynamometer and a pneumatic vigorimeter. Sarcopenia was defined using cut-offs from EWGSOP2, SDOC, and two DO-HEALTH-derived thresholds. Low muscle mass was identified using the fat-free mass index (FFMI) by BIA. Multivariate logistic regression was used to identify predictors of sarcopenia. Results: The prevalence of probable sarcopenia ranged from 68.1% to 89.4%, and confirmed sarcopenia from 39.6% to 50.3%, depending on the thresholds applied. Sarcopenic patients were older (86.1 ± 9.8 vs. 80.4 ± 11.0 years; *p* < 0.001), had lower BMI (20.7 ± 2.9 vs. 26.1 ± 4.8 kg/m^2^; *p* < 0.001), and were more frequently in long-term care (*p* = 0.014–0.043). Older age (OR 1.03–1.07 per year; *p* < 0.05) and lower BMI (OR 0.59–0.68 per kg/m^2^; *p* < 0.001) were independently associated with sarcopenia; sex and fall history were not. Conclusions: Sarcopenia prevalence was high and varied widely across diagnostic definitions and measurement tools, reflecting both methodological variability and the high vulnerability of hospitalized older adults. These findings highlight the need for standardized, context-adapted diagnostic strategies to guide timely intervention in high-risk hospitalized older adults.

## 1. Introduction

Sarcopenia, characterized by the progressive loss of muscle mass and strength, is a major health concern in older adults, leading to increased risks of falls, fractures, disability, hospitalizations, and mortality [1,2]. It is also a poor prognostic factor in conditions such as cancer [3], end-stage renal disease [4], diabetes [5], chronic obstructive pulmonary disease [6], and heart failure [7], where it contributes to disease progression, recurrent hospitalizations, and higher mortality [8,9,10]. The health-related quality of life (HRQoL) is also significantly reduced in sarcopenic patients [11]. In addition to its clinical burden, sarcopenia is associated with elevated healthcare costs due to higher hospitalization rates and medical expenses [12,13]. Despite these consequences, sarcopenia remains underdiagnosed in hospital settings, where acute illness and immobilization may further accelerate muscle loss, exacerbating its negative effects [14].

Recent European studies in hospitalized geriatric populations report sarcopenia prevalence ranging from 30% to 70%, depending on diagnostic criteria and measurement approach [1]. Beyond clinical measures, several circulating biomarkers have been linked to sarcopenia pathophysiology, including inflammatory markers (CRP, IL-6, TNF-α), nutritional indicators (albumin), and muscle-related factors such as myostatin, IGF-1, and the creatinine/cystatin C ratio. These reflect systemic inflammation, anabolic resistance, and metabolic stress that accelerate muscle catabolism. However, no single biomarker reliably defines sarcopenia, and their integration into diagnostic algorithms remains an area for ongoing research.

The European Working Group on Sarcopenia in Older People (EWGSOP2) has established criteria defining sarcopenia based on low muscle strength, confirmed by reduced muscle mass, and severe sarcopenia when functional impairment is present [15]. The dynamometer remains the reference instrument for maximal isometric handgrip strength (HGS) assessment and has been extensively validated in both community and hospital cohorts. The vigorimeter, which measures pneumatic pressure rather than traction force, offers an advantage in frail or cognitively impaired inpatients who may struggle to use a standard dynamometer. It has shown good reproducibility and bedside feasibility in geriatric wards [16,17]. The DO-HEALTH study established sex- and age-specific reference values for the vigorimeter [18], validated against physical performance outcomes in community-dwelling adults. However, these thresholds have not yet been formally validated in hospitalized populations.

Implementing HGS assessments in hospital settings necessitates adapting tools to diverse care environments [16,17]. For instance, in acute care, patients often face acute clinical instability, requiring brief and low-effort assessments. In rehabilitation, repeated HGS testing may help track recovery progress, while in long-term care, instruments must accommodate cognitive and functional limitations. Consistent use of the same HGS method ensures comparability across care settings, supports continuity of care, and facilitates international comparisons once reference values are validated.

Despite growing recognition of sarcopenia as a major determinant of adverse outcomes in hospitalized older adults, evidence comparing diagnostic definitions and measurement tools in this setting remains limited. Variability in handgrip strength assessment methods may substantially influence prevalence estimates and clinical interpretation. Therefore, this study aimed to compare the performance of dynamometer- and vigorimeter-based diagnostic definitions of sarcopenia in a real-world hospital cohort, addressing a key methodological gap in the literature. Specifically, we evaluated sarcopenia prevalence across four diagnostic criteria and examined its associations with patient characteristics and outcomes, including age, nutritional risk, falls, and hospitalization parameters. We hypothesized that sarcopenia prevalence would differ significantly depending on the selected diagnostic definition and measurement tool, with potential implications for screening strategies and clinical decision-making in acute geriatric care.

## 2. Materials and Methods

### 2.1. Design, Setting, and Population

We conducted a cross-sectional study in the Department of Rehabilitation and Geriatrics at Geneva University Hospitals (HUG) from 24 April to 22 June 2023. The department spans five specialized sites, comprising multiple wards: acute geriatrics (200 beds), acute internal medicine (36 beds), geriatric rehabilitation (128 beds), musculoskeletal rehabilitation (62 beds), internal medicine and oncology rehabilitation (83 beds), neurorehabilitation (19 beds), palliative care (36 beds), and long-term care for patients awaiting institutionalization (122 beds). All patients hospitalized during the study period were eligible for inclusion, regardless of age. This target population provided a census of all eligible inpatients during the study period, rather than a pre-specified sample size. Although no a priori power calculation was performed, the expected number of participants and outcome events was considered sufficient to support multivariable analyses, in accordance with the conventional ‘rule of ten,’ which recommends at least ten outcome events per predictor variable to ensure model stability and minimize overfitting. Exclusion criteria were end-of-life status, clinical instability, or upper limb motor neurological deficits.

The study protocol was submitted to the Geneva Cantonal Ethics Committee (Commission cantonale d’éthique de la recherche sur l’être humain, CCER), which granted an exemption from formal written consent as the study was classified as a quality improvement initiative. Approval was obtained before study initiation in March 2023. The study was conducted in accordance with the Declaration of Helsinki. Oral consent was obtained from all participants after being informed of the study procedures.

### 2.2. Data Collection

Over an eight-week period, two trained research assistants systematically screened all wards within the department for sarcopenia, following a predefined calendar. Each ward was visited once, ensuring every bed was assessed, and all patients were invited to participate. Oral consent was obtained after verbal explanation of the procedures.

For each participant, demographic and clinical data were collected from electronic medical records, including age, sex, ward type (categorized as acute care, rehabilitation, or long-term care), weight (measured within 24 h of the assessment), BMI, length of hospital stay, presence of in-hospital falls, and results from the Nutritional Risk Screening (NRS) and SARC-F questionnaire. The SARC-F questionnaire was initially included in the assessment protocol but was later omitted from analyses due to modifications in data collection. This rationale has been detailed in the Discussion. Height was measured during hospitalization whenever feasible, and the most recent medical-record value was used only when direct measurement was not possible. The NRS is a validated tool that evaluates nutritional status based on weight loss, intake, and disease severity; scores ≥ 3 indicate clinically relevant nutritional risk [19]. The SARC-F is a five-item tool assessing muscle function and fall risk, with total scores ranging from 0 to 10; a score ≥4 suggests a high likelihood of sarcopenia [20]. Although SARC-F was systematically administered, data were not included in this study’s analysis due to a shift in the study protocol toward universal objective screening.

### 2.3. Handgrip Strength Assessment

Handgrip strength (HGS) was measured using two validated instruments: the Jamar hydraulic dynamometer and the Martin pneumatic vigorimeter. For standardization, the medium-sized rubber bulb was used for all participants in the vigorimeter assessment. Each participant underwent four HGS assessments per instrument—two measurements per hand—starting with the dominant hand. A rest interval of 30 s was allowed between attempts. Participants were randomly assigned to start with one of the two tools using a predetermined number sequence (even = Jamar first; odd = vigorimeter first). During measurement, patients were seated upright, with shoulders adducted and neutrally rotated, elbows flexed at 90 degrees, and forearms and wrists in a neutral position.

The dynamometer is widely considered the gold standard for measuring maximal isometric handgrip strength. It operates through a hydraulic mechanism and provides a peak force reading in kilograms (kg) as the patient squeezes the rigid metal handle. This method reflects voluntary maximal contraction and primarily assesses the function of the extrinsic forearm muscles. In contrast, the vigorimeter consists of a soft rubber bulb connected to a manometer, capturing grip strength in kilopascals (kPa) based on the air pressure generated during sustained compression. This device may be more suitable for frail individuals, as it requires less initial force and allows better detection of deficits in hand and finger strength, coordination, and endurance. Its soft grip surface may also reduce discomfort in patients with arthritis or limited joint mobility.

To define low HGS, we applied two sex-specific cut-offs for each tool. For the dynamometer, thresholds from the European Working Group on Sarcopenia in Older People (EWGSOP2: <27 kg for men, <16 kg for women) and from the Sarcopenia Definitions and Outcomes Consortium (SDOC: <35.5 kg for men, <16 kg for women) were used [15,18]. For the Martin vigorimeter, two Swiss population-derived sets of cut-offs from the DO-HEALTH cohort were applied [21]. The first definition, HGS DO-HEALTH1, used the following thresholds: <64 kPa for men aged ≤75 years, <50 kPa for men aged >75 years, <42 kPa for women aged ≤75 years, and <34 kPa for women aged >75 years. The second definition, HGS DO-HEALTH2, applied slightly higher cut-offs: <69 kPa for men aged ≤75 years, <55 kPa for men aged >75 years, <46 kPa for women aged ≤75 years, and <39 kPa for women aged >75 years. These cut-offs were derived from a large, well-characterized cohort of community-dwelling older adults across Switzerland [21].

Following HGS assessment, muscle mass was measured by tetrapolar bioelectrical impedance analysis (BIA) using the Nutriguard MS device (Data Input GmbH, Pöcking, Germany). All measurements were conducted in the supine position, consistent with local protocol for hospitalized patients. Adhesive electrodes were placed on the dorsal surfaces of the right wrist, hand, ankle, and foot. A low-intensity alternating current (0.8 mA, 50 kHz) was passed through the body, and resistance and reactance were measured to estimate total fat-free mass using the Geneva formula, previously validated in this population [22]. The fat-free mass index (FFMI) was calculated by dividing fat-free mass by height squared (kg/m^2^). Low muscle mass was defined using the ESPEN-recommended cut-offs: FFMI < 17 kg/m^2^ for men and <15 kg/m^2^ for women [23].

### 2.4. Statistical Analysis

Descriptive statistics were used to summarize baseline characteristics of the study population. Continuous variables were presented as means with standard deviations, and categorical variables were expressed as percentages. Comparisons between groups (e.g., sarcopenia vs. non-sarcopenia, male vs. female) were performed using the Mann–Whitney u test for continuous variables and the chi-square test or Fisher’s exact test for categorical variables. The prevalence of sarcopenia was assessed using four different diagnostic criteria, including the EWGSOP2, SDOC, and DO-HEALTH cut-offs for handgrip strength (HGS) measured by the Jamar dynamometer and the Martin vigorimeter as described previously, as well as FFMI. A full multivariable logistic regression model was used to identify independent predictors of confirmed sarcopenia, with results reported as odds ratios (ORs) and 95% confidence intervals (CIs). Variance inflation factors (VIFs < 2.0) were examined for all predictor variables, confirming the absence of multicollinearity. Because the Jamar dynamometer (kg) and Martin vigorimeter (kPa) operate on different physical scales, no conversion equation was applied, and correlation was assessed visually using scatterplots. All tests were two-sided, with statistical significance set at *p* < 0.05. Analyses were conducted using Stata version 18.5.

## 3. Results

### 3.1. Study Population and Baseline Characteristic

**Figure 1:**
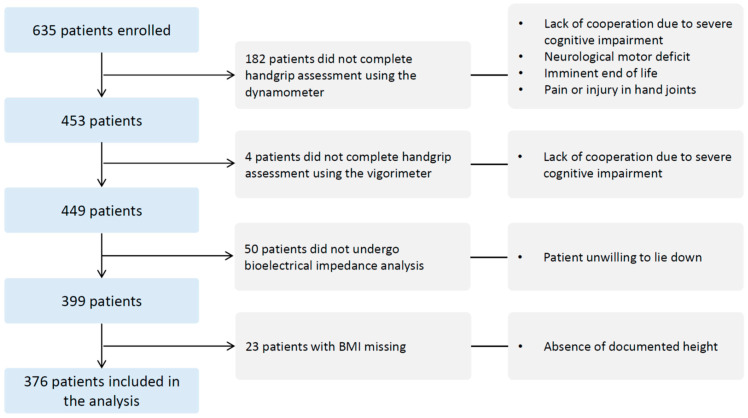
Flowchart of patient inclusion and exclusion for sarcopenia assessment.

Among the 635 patients hospitalized in the Department of Rehabilitation and Geriatrics during the study period, 376 were included in the final analysis. Figure 1 displays the participant flowchart, detailing exclusions due to clinical instability, end-of-life care, or upper limb neurological deficits precluding handgrip strength testing.

The characteristics of the study population are presented in Table 1. Overall, 56.6% of participants were female, and the mean age was 82.7 ± 10.9 years. The youngest patient was 43 years old, the oldest 101, with 92% aged ≥65 years and 82% ≥ 75 years. Patients aged ≥90 years represented 27.4% of the cohort, with women being significantly older than men (83.9 ± 10.8 vs. 81.1 ± 10.9 years; *p* = 0.0122). The population was distributed across long-term care units (16.5%), rehabilitation units (46.8%), and acute care units (36.7%).

The mean BMI was 24.0 kg/m^2^, with significant sex-based differences in distribution (*p* < 0.001).

**Figure 2:**
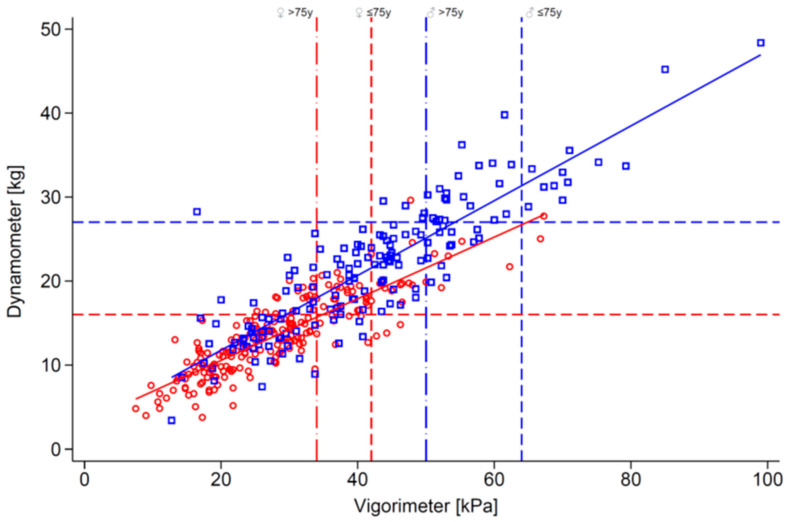
Scatterplots of handgrip strength values by sex using the dynamometer (kg, x-axis) and vigorimeter (kPa, y-axis). Dashed horizontal lines represent EWGSOP2 cut-offs for low HGS; vertical lines represent DO-HEALTH cut-offs. The plots illustrate the correlation between tools and the sex-based distribution of muscle strength. 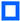 Male 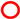 Female.

Figure 2 illustrates the correlation between dynamometer and vigorimeter values by sex, with overlaid diagnostic cut-offs. The plots highlight the concentration of female values near or below threshold lines, especially with the vigorimeter, and the broader dispersion among men.

Women were more frequently underweight (<18.5 kg/m^2^; 17.8% vs. 6.1%) or obese (>30 kg/m^2^; 14.1% vs. 6.1%), while men were more often in the normal or overweight categories. Nutritional risk (NRS-2002 score ≥ 3) was identified in 82.1% of patients, with no significant sex difference (*p* = 0.8982). Falls during hospitalization were common (45.2%) and occurred more frequently in men than women (52.1% vs. 39.9%; *p* = 0.0181). The SARC-F questionnaire, available for 200 patients, identified 69.0% as being at high risk of sarcopenia.

### 3.2. Muscle Strength Measures and Sarcopenia Prevalence According to Diagnostic Criteria

**Figure 3:**
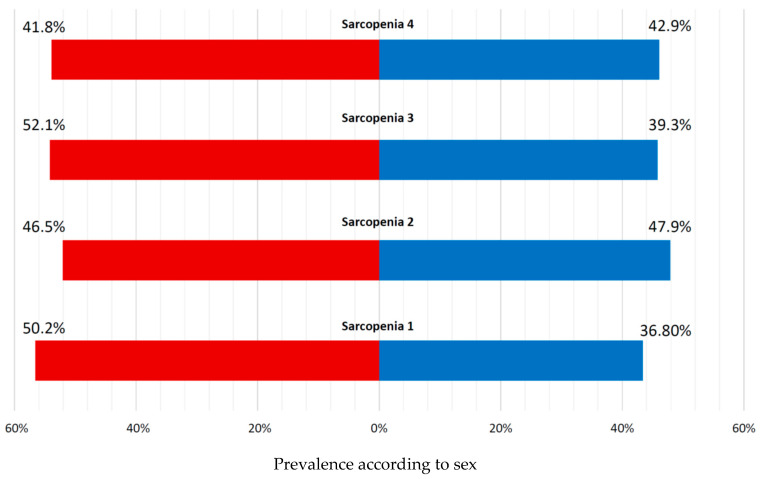
Prevalence of sarcopenia according to sex (women in red, men in blue). Sarcopenia 1 = HGS EWGSOP2 (<27 kg for men and <16 kg for women) and FFMI (<17 kg/m^2^ for men and <15 kg/m^2^ for women). Sarcopenia 2 = HGS SDOC (<35.5 kg for men and <16 kg for women) and FFMI (<17 kg/m^2^ for men and <15 kg/m^2^ for women). Sarcopenia 3 = HGS DO-HEALTH1 (<64 kPa for men ≤ 75 years, <42 kPa for women ≤ 75 years, <50 kPa for men > 75 years, <34 kPa for women > 75 years) and FFMI (<17 kg/m^2^ for men and <15 kg/m^2^ for women). Sarcopenia 4 = HGS DO-HEALTH2 (<69 kPa for men ≤ 75 years, <46 kPa for women ≤ 75 years, <55 kPa for men > 75 years, <39 kPa for women > 75 years) and FFMI (<17 kg/m^2^ for men and <15 kg/m^2^ for women). Abbreviations: HGS = handgrip strength; FFMI = fat-free mass index; EWGSOP2 = European Working Group on Sarcopenia in Older People; SDOC = Sarcopenia Definitions and Outcomes Consortium.

Figure 3 shows sarcopenia prevalence by diagnostic definition and sex. Using the Jamar dynamometer (Lafayette Instrument Company, Lafayette, IN, USA), 68.1% met EWGSOP2 criteria for probable sarcopenia, while 89.4% met SDOC criteria. The SDOC cut-offs identified significantly more men as sarcopenic (95.1% vs. 85.0%; *p* = 0.002), whereas EWGSOP2 criteria showed no sex difference (*p* = 0.262). Using the Martin vigorimeter (KLS Martin SE & Co. KG, Tuttlingen, Germany), probable sarcopenia prevalence was 72.6% and 84.0% according to the two DO-HEALTH cut-offs, with no significant sex differences (*p* = 0.700 and *p* = 0.774, respectively).

**Figure 4:**
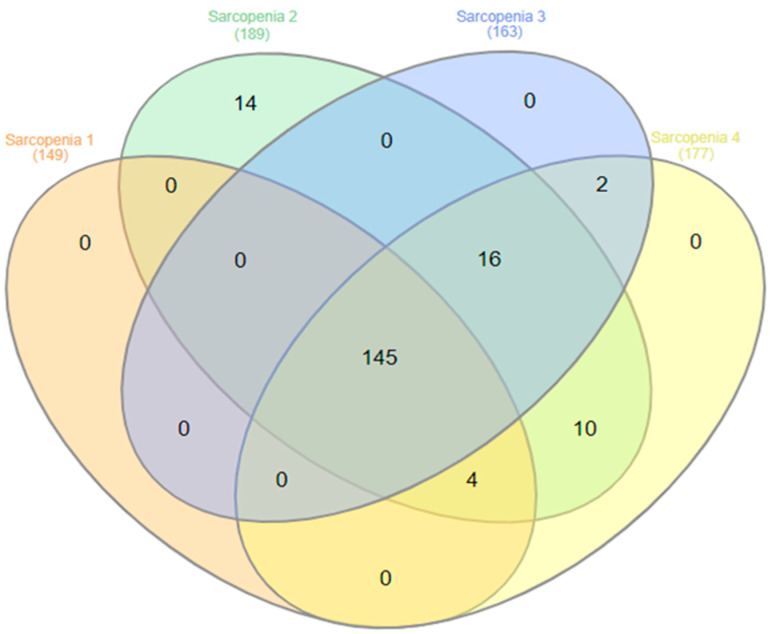
Venn diagram analysis showing the overlap of diagnostic criteria for sarcopenia (number of cases). **Sarcopenia 1** = HGS EWGSOP2 (<27 kg for men and <16 kg for women) and FFMI (<17 kg/m^2^ for men and <15 kg/m^2^ for women). **Sarcopenia 2** = HGS SDOC (<35.5 kg for men and <16 kg for women) and FFMI (<17 kg/m^2^ for men and <15 kg/m^2^ for women). **Sarcopenia 3** = HGS DO-HEALTH1 (<64 kPa for men ≤ 75 years, <42 kPa for women ≤ 75 years, <50 kPa for men > 75 years, <34 kPa for women > 75 years) and FFMI (<17 kg/m^2^ for men and <15 kg/m^2^ for women). **Sarcopenia 4** = HGS DO-HEALTH2 (<69 kPa for men ≤ 75 years, <46 kPa for women ≤ 75 years, <55 kPa for men > 75 years, <39 kPa for women > 75 years) and FFMI (<17 kg/m^2^ for men and <15 kg/m^2^ for women). Abbreviations: HGS = handgrip strength; FFMI = fat-free mass index; EWGSOP2 = European Working Group on Sarcopenia in Older People; SDOC = Sarcopenia Definitions and Outcomes Consortium.

Confirmed sarcopenia (low HGS and low FFMI) ranged from 39.6% to 50.3%, depending on the criteria. Although detailed comorbidity indices were not collected, the high prevalence of sarcopenia observed likely reflects the underlying vulnerability of this population and the physiological stress associated with acute illnesses. Low FFMI, defined by ESPEN cut-offs, was observed in 53.5% of patients, with no significant sex difference (*p* = 0.090; see Table 2). Figure 4 shows partial but incomplete overlap between diagnostic definitions, indicating that varying thresholds and instruments identify overlapping yet distinct subsets of patients.

Table 3 compares patients with and without sarcopenia per EWGSOP2 criteria. Sarcopenic patients were older (86.1 ± 9.8 vs. 80.4 ± 11.0 years; *p* < 0.001), with 40.3% aged ≥90 years. They also had significantly lower body weight (55.0 ± 10.4 kg vs. 70.8 ± 13.8 kg; *p* < 0.001) and BMI (20.7 ± 2.9 vs. 26.1 ± 4.8 kg/m^2^; *p* < 0.001). Underweight BMI was more frequent among sarcopenic patients (24.2% vs. 5.3%; *p* < 0.001). No cases of sarcopenic obesity were identified.

Sarcopenic patients had higher nutritional risk (NRS-2002 score 4.2 ± 1.1 vs. 3.4 ± 1.2; *p* < 0.001); 90.3% were at risk of malnutrition, versus 76.5% of non-sarcopenic patients (*p* = 0.0072). Falls were also more common in the sarcopenic group (52.3% vs. 40.5%; *p* = 0.0243). No significant differences in sarcopenia prevalence were observed across care levels (*p* = 0.1424).

### 3.3. Clinical Correlates and Predictors of Sarcopenia

Multivariate logistic regression models (Table 4) identified age and BMI as the most consistent predictors of sarcopenia. Each additional year of age increased sarcopenia odds (OR 1.03–1.07; *p* < 0.05), while higher BMI was protective (OR 0.59–0.68; *p* < 0.001).

Compared to acute care, long-term care was associated with increased sarcopenia risk under Sarcopenia 1 (OR 2.70, 95% CI 1.23–5.96, *p* = 0.014) and Sarcopenia 4 (OR 2.28, 95% CI 1.03–5.05, *p* = 0.043). Rehabilitation care showed a non-significant trend toward higher sarcopenia prevalence. Falls during hospitalization and sex were not independently associated with sarcopenia in any model (*p* = 0.083 and *p* = 0.27–0.87, respectively).

An exploratory model including the NRS score did not alter core associations, but reduced sample size due to missing data weakened power. Therefore, NRS was excluded from final models to retain the full cohort (N = 376). The final models explained 32–42% of sarcopenia status variance, suggesting that additional variables such as comorbidities, inflammation, or function may enhance future predictive models.

## 4. Discussion

This study highlights the high prevalence of probable sarcopenia in hospitalized older adults, with probable sarcopenia affecting 68.1% to 89.4% of patients, depending on the diagnostic criteria used. Confirmed sarcopenia was also highly prevalent, ranging from 39.6% to 50.3%. The strongest predictors of sarcopenia were older age, lower BMI, and long-term care admissions, emphasizing the need for systematic screening and tailored interventions in hospital settings.

The prevalence reported in our study is notably higher than in community-based populations. For example, Bertschi et al. found a probable sarcopenia prevalence of 24.6% in a Swiss geriatric population using similar vigorimeter-based DO-HEALTH cut-offs [22]. Similarly, the prevalence of confirmed sarcopenia, defined using EWGSOP2 handgrip strength cut-offs and ESPEN fat-free mass index (FFMI) criteria, was 39.6% in our study—almost twice as high as the 22.6% reported in the same Swiss cohort [1]. Our findings align more closely with studies conducted in institutionalized populations, such as the SENIOR study in Belgian nursing home residents, where sarcopenia prevalence reached 38.1% using EWGSOP criteria [24]. These results suggest that hospitalized older adults are at a significantly higher risk of sarcopenia than previously estimated in community-dwelling individuals.

The wide range of prevalence observed across prior hospital-based studies (7.2% to 73%) likely reflects variation in diagnostic methods and patient characteristics [15,25,26,27,28,29]. Our results confirm that hospitalization represents a critical window of vulnerability for muscle loss. In our multivariate analysis, the long-term care setting was independently associated with sarcopenia, which may reflect cumulative effects of chronic frailty, malnutrition, physical inactivity, and institutional dependency in these patients. This finding underscores the importance of proactive sarcopenia screening and intervention even outside acute care [30].

Furthermore, hospitalization concentrates multiple pathways that accelerate muscle loss beyond age alone. Acute physiological stress and inactivity (even brief bed rest) rapidly reduce lean mass and strength in older adults, with ~10 days of immobilization producing marked decrements in muscle function and capacity, highlighting hospital-associated deconditioning as a key driver [31]. Concurrent inflammatory activation (elevated IL-6/CRP) promotes proteolysis and suppresses synthesis, while age-related anabolic resistance blunts the muscle protein synthetic response to nutrients and exercise—together amplifying catabolism during acute illness [32].

Additionally, the choice of diagnostic thresholds had a major impact: the SDOC criteria classified almost 90% of patients as sarcopenic, compared to 68.1% using EWGSOP2. These differences highlight the need for context-adapted thresholds in hospitalized populations. Importantly, this study compared two different handgrip strength instruments—the dynamometer and the vigorimeter. While the dynamometer remains the gold standard in many guidelines, the vigorimeter may offer practical advantages in frail or cognitively impaired patients due to its lower resistance, ease of use, and greater sensitivity to submaximal effort. The inclusion of both tools allowed us to explore their application across diverse hospital settings. Consistent and adaptable measurement approaches are key for real-world screening programs.

From a clinical perspective, early identification of sarcopenia offers a critical window for targeted intervention across the continuum of care. In acute hospital settings, handgrip strength (HGS) represents a rapid, low-cost, and non-invasive screening tool that can flag patients at high nutritional and functional risk at admission. In rehabilitation units, repeated HGS measurements provide a simple means to monitor treatment response and recovery trajectories, while in long-term care, periodic assessment may assist in tracking progressive decline and guiding individualized care planning. Because our cross-sectional design does not allow differentiation between pre-existing muscle loss and acute decompensation, the relative contribution of chronic versus acute (“acute-on-chronic”) sarcopenia remains uncertain. Longitudinal studies are needed to characterize these trajectories and assess their reversibility under targeted interventions [32,33].

In high-risk inpatients, the detection of probable sarcopenia based on low HGS alone may already warrant prompt initiation of nutritional and physiotherapy measures, given their favorable risk–benefit profile. Evidence from the EFFORT trial and related inpatient cohorts indicates that baseline HGS predicts complications, mortality, and response to nutritional support [33,34]. Future work should determine whether confirmatory testing adds meaningful clinical value or whether acting on probable sarcopenia provides a cost-effective strategy to improve outcomes in hospitalized older adults.

This study has several limitations. Its cross-sectional design precludes causal inference and limits the ability to determine whether sarcopenia preceded hospitalization or developed secondary to acute illness. The exclusion of the frailest patients—those with severe cognitive impairment or unable to complete assessments—likely led to conservative prevalence estimates of the true burden of sarcopenia in a hospitalized population. Bioelectrical impedance analysis (BIA), although practical in acute care, is less precise than DXA and sensitive to hydration status, which was not standardized; however, the use of a locally validated Geneva-specific equation supports internal validity. The DO-HEALTH vigorimeter cut-offs, while the most robust currently available, were derived from community-dwelling older adults and require further validation in hospitalized populations. Because the dynamometer and vigorimeter measure different physical constructs—traction force (kg) versus pneumatic pressure (kPa)—and no validated conversion equation exists between them, the Bland–Altman analysis was not appropriate. Prior studies have confirmed their non-interchangeability [35,36]. Finally, the single-center design and absence of inflammatory biomarkers limit generalizability and mechanistic interpretation.

## 5. Conclusions

In conclusion, sarcopenia is highly prevalent among hospitalized older adults, particularly in those with advanced age, low BMI, and nutritional risk. Our study provides novel evidence comparing different diagnostic thresholds and instruments in this setting. Hospitalization represents a critical period for accelerated muscle decline, reinforcing the need for systematic sarcopenia screening. Future research should define optimal cut-offs and integrate sarcopenia screening into hospital care pathways to enable timely, individualized interventions.

## Figures and Tables

**Table 1:** Characteristics of the study population.

Characteristics	Female	Male	Total	*p*-Value
N	213 (56.6%)	163 (43.4%)	376 (100.0%)	
Age, y	83.9 (10.8)	81.1 (10.9)	82.7 (10.9)	0.0122
Age category, *n* (%)				
<70	21 (9.9%)	25 (15.3%)	46 (12.2%)	0.0763
70–79.9	40 (18.8%)	38 (23.3%)	78 (20.7%)	
80–89.9	84 (39.4%)	65 (39.9%)	149 (39.6%)	
90+	68 (31.9%)	35 (21.5%)	103 (27.4%)	
Level of care, *n* (%)				
Long-term	31 (14.6%)	31 (19.0%)	62 (16.5%)	0.4620
Rehabilitation	104 (48.8%)	72 (44.2%)	176 (46.8%)	
Acute	78 (36.6%)	60 (36.8%)	138 (36.7%)	
Length of stay, day	56.9 (79.9)	72.7 (119.1)	63.7 (98.9)	0.1259
Weight, kg	60.2 (14.9)	70.2 (12.3)	64.6 (14.7)	<0.0010
Height, cm	158.4 (7.2)	171.1 (6.8)	163.9 (9.5)	<0.0010
BMI, kg/m^2^	24.0 (5.7)	24.0 (3.8)	24.0 (5.0)	0.9340
BMI category, *n* (%)				
<18.5	38 (17.8%)	10 (6.1%)	48 (12.8%)	<0.0010
18.5–24.9	92 (43.2%)	88 (54.0%)	180 (47.9%)	
25–29.9	53 (24.9%)	55 (33.7%)	108 (28.7%)	
≥30	30 (14.1%)	10 (6.1%)	40 (10.6%)	
NRS 0–7 (N = 229)	3.7 (1.2)	3.7 (1.2)	3.7 (1.2)	0.8880
NRS ≥ 3, *n* (%)	108 (81.8%)	80 (82.5%)	188 (82.1%)	0.8982
Fall during hospitalization	85 (39.9%)	85 (52.1%)	170 (45.2%)	0.0181
SARC-F, *n* (%) N = 200				
Normal	28 (26.7%)	34 (35.8%)	62 (31.0%)	0.1636
Sarcopenia risk	77 (73.3%)	61 (64.2%)	138 (69.0%)	
Jamar dynamometer, kg	13.7 (4.6)	21.4 (7.6)	17.0 (7.2)	<0.0010
Martin vigorimeter, kPa	28.6 (10.8)	41.7 (14.8)	34.3 (14.2)	<0.0010
FFMI, kg/m^2^	15.0 (2.8)	17.2 (2.4)	15.9 (2.8)	<0.0010
FMI, kg/m^2^	9.0 (3.7)	6.7 (2.5)	8.0 (3.5)	<0.0010

Abbreviations: BMI = body mass index; NRS = nutritional risk screening; SARC-F = Strength, Assistance Walking, Rise From a Chair, Climb Stairs, and Falls Questionnaire; FFMI = fat-free mass index; FMI = fat mass index.

**Table 2:** Prevalence of low fat-free mass index and handgrip strength according to sex.

FFMI	Female	Male	Total	*p* Value
Below	122 (57.3%)	79 (48.5%)	201 (53.5%)	0.090
Normal	91 (42.7%)	84 (51.5%)	175 (46.5%)	
**Jamar dynamometer** **(EWGSOP2)**				
Below	140 (65.7%)	116 (71.2%)	256 (68.1%)	0.262
Normal	73 (34.3%)	47 (28.8%)	120 (31.9%)	
**Jamar dynamometer** **(SDOC)**				
Below	181 (85.0%)	155 (95.1%)	336 (89.4%)	0.002
Normal	32 (15.0%)	8 (4.9%)	40 (10.6%)	
**Martin vigorimeter** **(DO-HEALTH1)**				
Below	153 (71.8%)	120 (73.6%)	273 (72.6%)	0.700
Normal	60 (28.2%)	43 (26.4%)	103 (27.4%)	
**Martin vigorimeter** **(DO-HEALTH2)**				
Below	178 (83.6%)	138 (84.7%)	316 (84.0%)	0.774
Normal	35 (16.4%)	25 (15.3%)	60 (16.0%)	

Abbreviations: FFMI = fat-free mass index; EWGSOP2 = European Working Group on Sarcopenia in Older People; SDOC = Sarcopenia Definitions and Outcomes Consortium. DO-HEALTH1: <64 kPa for men ≤ 75 years, <42 kPa for women ≤ 75 years, <50 kPa for men > 75 years, <34 kPa for women > 75 years. DO-HEALTH2: <69 kPa for men ≤ 75 years, <46 kPa for women ≤ 75 years, <55 kPa for men > 75 years, <39 kPa for women > 75 years.

**Table 3:** Comparison of patients with and without sarcopenia.

Characteristics	Sarcopenia 1			
	No	Yes	Total	*p* Value
N	227 (60.4%)	149 (39.6%)	376 (100.0%)	
Age, y	80.4 ± 11.0	86.1 ± 9.8	82.7 ± 10.9	<0.0010
Age category, *n* (%)				
<70	37 (16.3%)	9 (6.0%)	46 (12.2%)	<0.0010
70–79.9	56 (24.7%)	22 (14.8%)	78 (20.7%)	
80–89.9	91 (40.1%)	58 (38.9%)	149 (39.6%)	
90+	43 (18.9%)	60 (40.3%)	103 (27.4%)	
Female sex, *n* (%)	124 (54.6%)	89 (59.7%)	213 (56.6%)	0.3285
Level of care, *n* (%)				
Long-term	32 (14.1%)	30 (20.1%)	62 (16.5%)	0.1424
Rehabilitation	104 (45.8%)	72 (48.3%)	176 (46.8%)	
Acute	91 (40.1%)	47 (31.5%)	138 (36.7%)	
Length of stay, day	56.5 ± 84.4	74.6 ± 117.0	63.7 ± 98.9	0.0843
Weight, kg	70.8 ± 13.8	55.0 ± 10.4	64.6 ± 14.7	<0.0010
Height, cm	164.7 ± 9.0	162.7 ± 10.0	163.9 ± 9.5	0.0491
BMI (kg/m^2^)	26.1 ± 4.8	20.7 ± 2.9	24.0 ± 5.0	<0.0010
BMI category, *n* (%)				
<18.5	12 (5.3%)	36 (24.2%)	48 (12.8%)	<0.0010
18.5–24.9	77 (33.9%)	103 (69.1%)	180 (47.9%)	
25–29.9	98 (43.2%)	10 (6.7%)	108 (28.7%)	
≥30	40 (17.6%)	0 (0.0%)	40 (10.6%)	
NRS 0–7 (N = 229)	3.4 ± 1.2	4.2 ± 1.1	3.7 ± 1.2	<0.0010
NRS ≥ 3, *n* (%)	104 (76.5%)	84 (90.3%)	188 (82.1%)	0.0072
Fall during hospitalization	92 (40.5%)	78 (52.3%)	170 (45.2%)	0.0243
SARC-F, *n* (%) N = 200				
Normal	40 (34.2%)	22 (26.5%)	62 (31.0%)	0.2471
Sarcopenia risk	77 (65.8%)	61 (73.5%)	138 (69.0%)	

Abbreviations: BMI = body mass index; NRS = nutritional risk screening; SARC-F = Strength, Assistance Walking, Rise From a Chair, Climb Stairs, and Falls Questionnaire. Sarcopenia 1 = HGS EWGSOP2 (<27 kg for men and <16 kg for women) and FFMI (<17 kg/m^2^ for men and <15 kg/m^2^ for women).

**Table 4:** Multivariate logistic regression models for factors associated with sarcopenia.

Characteristics	Sarcopenia 1				Sarcopenia 2				Sarcopenia 3				Sarcopenia 4			
	OR Adj	95% CI	*p*-Value	R^2^	OR Adj	95% CI	*p*-Value	R^2^	OR Adj	95% CI	*p*-Value	R^2^	OR Adj	95% CI	*p*-Value	R^2^
Male sex	1.05	(0.61–1.80)	0.870	32%	0.89	(0.50–1.58)	0.692	42%	0.80	(0.47–1.35)	0.399	32%	0.74	(0.43–1.28)	0.276	37%
Age	1.07	(1.04–1.10)	<0.001		1.04	(1.01–1.06)	0.012		1.04	(1.01–1.06)	0.006		1.03	(1.00–1.06)	0.030	
BMI	0.68	(0.63–0.74)	<0.001		0.59	(0.52–0.65)	<0.001		0.67	(0.62–0.73)	<0.001		0.64	(0.58–0.70)	<0.001	
In-hospital fall during	1.39	(0.81–2.36)	0.229		1.62	(0.90–2.91)	0.107		1.48	(0.87–2.52)	0.144		1.63	(0.94–2.84)	0.083	
Level of care																
Acute	1.00				1.00				1.00				1.00			
Long-term	2.70	(1.23–5.96)	0.014		1.86	(0.81–4.29)	0.144		1.95	(0.90–4.22)	0.088		2.28	(1.03–5.05)	0.043	
Rehabilitation	1.70	(0.94–3.09)	0.078		1.42	(0.75–2.69)	0.284		1.46	(0.81–2.61)	0.207		1.72	(0.93–3.15)	0.082	

**Sarcopenia 1** = HGS EWGSOP2 (<27 kg for men and <16 kg for women) and FFMI (<17 kg/m^2^ for men and <15 kg/m^2^ for women). **Sarcopenia 2** = HGS SDOC (<35.5 kg for men and <16 kg for women) and FFMI (<17 kg/m^2^ for men and <15 kg/m^2^ for women). **Sarcopenia 3** = HGS DO-HEALTH1 (<64 kPa for men ≤ 75 years, <42 kPa for women ≤ 75 years, <50 kPa for men > 75 years, <34 kPa for women > 75 years) and FFMI (<17 kg/m^2^ for men and <15 kg/m^2^ for women). **Sarcopenia 4** = HGS DO-HEALTH2 (<69 kPa for men ≤ 75 years, <46 kPa for women ≤ 75 years, <55 kPa for men > 75 years, <39 kPa for women > 75 years) and FFMI (<17 kg/m^2^ for men and <15 kg/m^2^ for women). Abbreviations: BMI = body mass index.

## Data Availability

The datasets generated and analyzed during the current study are not publicly available due to ethical and institutional restrictions, but are available from the corresponding author upon reasonable request.
